# Evaluation of novel inactivated vaccines for the SAT 1, SAT 2 and SAT 3 serotypes of foot-and-mouth disease in pigs

**DOI:** 10.1186/s12985-019-1262-1

**Published:** 2019-12-16

**Authors:** Hye-Eun Jo, Su-Hwa You, Joo-Hyung Choi, Mi-Kyeong Ko, Sung Ho Shin, Jisoo Song, Hyundong Jo, Min Ja Lee, Su-Mi Kim, Byounghan Kim, Jong-Hyeon Park

**Affiliations:** Center for Foot-and-Mouth Disease Vaccine Research, Animal and Plant Quarantine Agency, 177 Hyeoksin 8-ro, Gimcheon, Gyeongsangbuk-do Republic of Korea

**Keywords:** FMD, Vaccine, SAT1, SAT2, SAT3, Pig, Protection

## Abstract

**Background:**

The foot-and-mouth disease (FMD) virus is classified into seven serotypes, of which the South African types have South African Territories (SAT)1, SAT2, and SAT3 that are prevalent in Africa. Especially SAT2 have spread to Arabian Peninsula and the Palestinian Autonomous Territories. Of these viruses, the incidence of SAT2 is the highest. It is important to prepare for the spread of the virus to other continents, even though most FMD viruses are bovine-derived. In particular, due to the high breeding density of pigs in Asia, more attention is usually paid to the immunity and protection of pigs than cattle. For this reason, this study investigated the immunity and protection of pigs against the SAT viruses.

**Methods:**

Specific vaccines were developed for SAT1, SAT2, and SAT3 serotypes. These vaccine viruses were designed to be distinguished from the wild-type strain. An immunogenicity test was conducted using these vaccines in both cattle (*n* = 5/group) and pigs (*n* = 20/group).

**Results:**

High virus-neutralizing titer of antibodies (> 1:100) was induced in only 2 weeks after the immunization of cattle with the individual vaccine for SAT1, SAT2 or SAT3, and a clear immune response was induced after the second immunization in pigs. When the vaccinated pigs (*n* = 4–5/group) were challenged by the homologous wild-type virus strain 4 weeks after immunization, all the pigs were protected from the challenge.

**Conclusions:**

This study confirmed that these vaccines can be used against SAT1, SAT2, and SAT3 viruses in cattle and pigs. The vaccine strains developed in this study are expected to be used as vaccines that can protect against FMD in the event of a future FMD outbreak in pigs in consideration of the situation in Asia.

## Highlights

1. The cattle and pigs immunized with 1 ml of vaccines produced sufficient neutralizing antibodies for protection against FMD
2. The pigs vaccinated with SAT type vaccines were protected from the challenge of the wild-type virus.

## Background

The foot-and-mouth disease virus (FMDV) affects livestock and the trade for animal products globally. It is a contagious viral vesicular disease that affects cloven-hoofed animals. The disease has an economic impact and disrupts international trade in the livestock industry [1]. Although mortality caused by the FMD in infected animals is low, outbreaks result in significant economic consequences due to direct losses—such as low milk and meat production, treatment cost—as well as trade limitations in animal and animal products. The FMDV is classified as an *Aphthovius* genus of the *Picornaviridae* family. Seven serotypes of FMDV have been identified—A, O, C, Asia1, SAT1, SAT2, and SAT3. There is no cross-protection and immunity between the different serotypes [2], and effective vaccines must match the subtypes that are circulating in the field.

The SAT1, SAT2, and SAT3 viruses were first identified in the 1940s [3, 4]. All three types are confined to sub-Saharan Africa and affect mainly ruminants, although the prevalence of SAT1 (1961–1965 and 1970) and SAT2 (1990 and 2000) viruses have been recorded in the Middle East [5, 6]. Also, incursions into North Africa and the Middle East have also been recorded in recent years. Since 2012, FMDV outbreaks of SAT2 have been reported in Egypt, Libya, and the Palestinian Autonomous Territories. The outbreak of the FMD SAT2 virus in Egypt in 2012 was the first known occurrence of this serotype in the country since 1950 [7]. Outbreaks of SAT topotype viruses have been associated with transmission to livestock from wild animals, and African buffalo-mediated transmission has been confirmed in South and West Africa [8, 9]. Most of the viruses reported in these areas are the SAT2 type viruses; the SAT2-mediated outbreak is rarely reported in pigs [10]. Nevertheless, only the SAT2 vaccine has been partially evaluated in pigs [1, 11].

It is necessary to prepare for situations where vaccines are needed urgently in the absence of the FMD outbreak. Pork accounts for more than one-third of meat produced worldwide. Currently, pig production is an important component of food security and agricultural economies in Asia. Based on genetic and antigenic analyses, FMDVs throughout the world have been subdivided into seven regional pools. FMD outbreaks result from the spreading of the FMDV originating from pool 2 and subsequent mixing with the virus originating from pool 1 [12]. The vaccine immunity in pigs was revealed to be lower than that in cattle. This is a very worrisome phenomenon even for viruses that are endemic to Africa, compared with the spreading patterns of FMD.

The Korean vaccine policy has been switched to a national vaccination policy since 2011 [13, 14], and cattle and pigs are currently vaccinated against O and A types [15]. As trade and travel become more frequent, the risk of virus transmission is increasing. In order to build an antigen bank so that candidate vaccine strains can be developed promptly and used in emergencies in preparation for the influx of FMDV serotypes—of which outbreak has never been reported—viruses that express the capsid-encoding regions of SAT1 BOT 1/68 (topotype III), SAT2 ZIM 5/81 (topotype II), and SAT3 ZIM 4/81 (topotype I) strains have been developed. Thus, this study aimed to evaluate the immunogenicity and protection ability of the inactivated vaccines that contain the antigens produced by the vaccine strains in cattle and pigs, as described above.

## Materials and methods

### Cells, viruses, and plasmids

To create chimeric SAT-type viruses, P1 of O1 Manisa was replaced, in which the plasmid containing the O1 Manisa virus genome—which was established by replacing the 3B_1_B_2_ region with the 3B_3_B_3_ region, as described in the previous study [16]—was used. At the same time, an infectious clone was also used, in which the 142nd residue was changed from C to T (C142T) at the 3C region. Polymerase chain reaction (PCR) primers used for synthesizing cDNAs for each of the three SAT serotypes SAT1 BOT 1/68 (AY593845), SAT2 ZIM 5/81 (EF134951), and SAT3 ZIM 4/81 (KX375417) as well as for specifically amplifying the P1 genes are described in Table 1.

**Table 1:** The primers used for PCR to replace the P1 genes of three serotypes in pO Manisa 3B3C (p3B3C) template

Serotype	Direction	Primer sequences
SAT1-BOT	Forward	5′-AAGGTCCAGAAAAGGCTCAAGGGAGCAGGCCAGTCGTCACCA-3’
	Reverse	5′-GAGCAGGTCAAAATTAGAAGCTGTTTGGCAGGTTTAACAAG-3’
SAT2-ZIM	Forward	5′-AACAAAGGTCCAGAAAAGGCTCAAGGGAGCGGGACAGTCATCA-3’
	Reverse	5′-TTTGAGCAGGTCAAATTTAGAAGTGCACAGTTGTTTCTCGACG-3’
SAT3-ZIM	Forward	5′-AAACAAAGGTCAGAAAAGGCTCAAGGGAGCAGGCCAATCCTCCC-3’
	Reverse	5′-TTTGAGCAGGTCAAAATTTAGAAGTTTGTTTGTCAGGTGCAACCA-3’

The following are the PCR conditions for the amplification of the P1 genes: a mixture of 5X buffer (FINNZYMES, 10 μl), 10 mM dNTPs (1 μl), Phusion enzyme (1 μl of 2 U/μl), and sterile distilled water (35 μl) was reacted at 98 °C for 30 s, 25 cycles at 98 °C for 10 s, 72 °C for 30 s, 72 °C for 2 min 30 s, and finally at 72 °C for 10 min. The amplified P1 PCR product was reacted according to the ligation conditions provided by the Gibson Assembly® Cloning Kit.

**Fig. 1:**
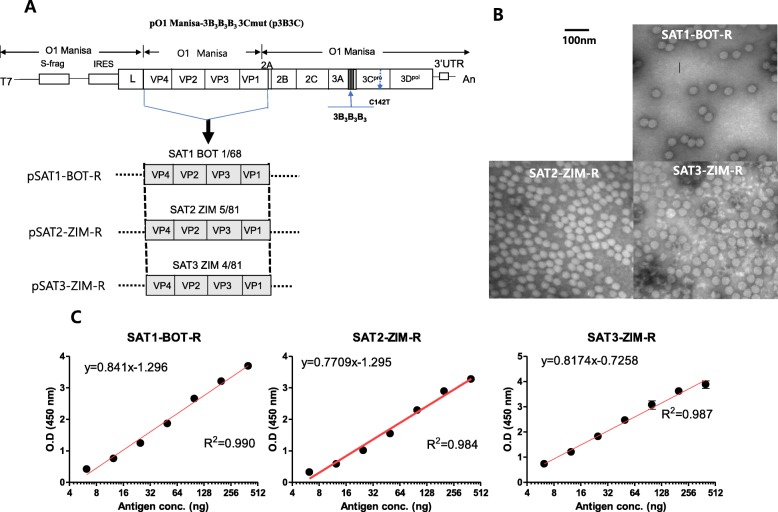
Characteristics of the chimeric foot-and-mouth disease virus with SAT viruses P1 (VP1–4), 3B and 3C mutation. **a** Schematic depiction of the chimeric viruses expressing structural proteins of SAT1, SAT2, and SAT3 viruses: SAT1 BOT 1/68 (AY593845), SAT2 ZIM 5/81 (EF134951), and SAT3 ZIM 4/81 (KX375417). The infectious cDNA clone is O1 Manisa, with a format of 3B_3_B_3_B_3_ in the 3B region and C142T in the 3C region. **b** Images of chimeric virus particles (SAT1 BOT-R, SAT2-ZIM-R, and SAT3-ZIM-R) via electron microscopy. The bar represents 100 nm. **c** Identification of recombinant FMDV structural proteins (SAT1, SAT2, SAT3) using antigen ELISA

For the PCR performed to amplify only the gene part—excluding P1 of the three FMD SAT serotypes using the infectious plasmid template (pO-Manisa 3B_3_B_3_B_3_ 3Cmut: p3B3C)—template p3B3C (100 ng/μl, 1 μl) DNA, primer VF sense 5′-ACTTCTAAATTTTGACCTGC-3′, and primer VR antisense 5′-CTTGAGCCTTTTCTGGAC-3′ were used (Fig. 1a). In this regard, the following were the PCR conditions: a mixture of 5X buffer (FINNZYMES, 10 μl), 10 mM dNTPs (1 μl), Phusion enzyme (1 μl of 2 U/μl), and sterile distilled water (35 μl) was reacted at 98 °C for 30 s, 25 cycles at 98 °C for 10 s, 65 °C for 30 s, 72 °C for 2 min 30 s, and finally at 72 °C for 10 min. To replace the SAT P1 into the p3B3C vector (P1-deleted linearized p3B3C), where P1 in the vector was excluded by PCR, the P1 PCR product (200 ng/μl, 1 μl) and the prepared p3B3C vector (P1-deleted linearized p3B3C) were reacted with the Gibson Assembly Master Mix (2X, 10 μl) and sterile distilled water (8 μl) at 56 °C for 30 min. Subsequently, the reaction mixture was transformed into the *E.coli* competent cells included in the Gibson Assembly® Cloning Kit. Finally, the DNA of the obtained clones was sequenced to confirm whether the P1 in the p3B3C plasmid was replaced correctly with the P1 of the three SAT serotypes—SAT1 BOT 1/68, SAT2 ZIM 5/81, and SAT3 ZIM 4/81 strains.

### Virus recovery and cell culture

ZZ-R and BHK-21 cells were maintained as previously described [17]. FMD viruses were isolated from the infectious clones according to a previous experimental method [18]. The P1-inserted recombinant plasmids (pO1m SAT1 BOT 1/68-R, pO1m SAT2 ZIM 5/81-R, and pO1m SAT3 ZIM 4/81- R) were digested with *Spe* l, a restriction enzyme (NEB, Beverly, USA), and then incubated at 37 °C for 24 h to create a single fragment of the gene. The purified DNA was transfected into BHK/T7–9 cells (T7 RNA polymerase-expressing baby hamster kidney cell line) with lipofectamine 2000 (Invitrogen, Carlsbad, USA), and the cells were incubated for 2~3 days; then, the P1-inserted FMD viruses (SAT1 BOT 1/68-R, SAT2 ZIM 5/81-R, and SAT3 ZIM 4/81-R) were obtained. Next, viruses were amplified through at least five consecutive passages using ZZ-R (fetal goat tongue epithelium cell) cells or BHK-21 (baby hamster kidney) cells.

Further, to produce antigens for inactivated vaccine production, viruses were amplified using BHK21 or BHK21-suspension cells, which are FMD-antigen-producing cells. Sixteen hours after infection, the viruses were inactivated by 0.003 N of binary ethylenimine (BEI) for 24 h at 26 °C and concentrated with polyethylene glycol 6000 (Sigma Aldrich, WI, USA). The virus was layered on 15%~ 45% sucrose-density gradients and centrifuged. After ultracentrifugation, the bottom of the centrifuge tube was punctured, and 1 ml fractions were collected. As done in a previous study [17], the final inactivated antigen (FMD viral particles) were quantified for determination of vaccine dose and tested using transmission electron microscopy for virus particle examination. For the antigen ELISA, the 96-well flat-bottom plate was coated with 100 μl/well of each two-fold diluted antigen (6–512 ng) 4 °C overnight. The plates were washed three times with 200 μl PBST. The positive reference antiserum (type-specific monoclonal antibodies) was added to the wells and incubated for 1 h at 37 °C. The plates were washed three times with 200 μl PBST and were added goat-anti mouse IgG (100 ng/ml) conjugated to HRP with 100 μl and incubated for 1 h at 37 °C. The plates were washed three times with 200 μl PBST. One hundred μl of 3,3′,5,5′-tetramethyl -benzidine (TMB) substrate was added to the plates, which were then incubated in the dark for 15 min at room temperature. The reaction was stopped with 50 μl H_2_SO_4_, and the optical density (OD) was measured with an ELISA reader at 450 nm.

### Vaccine formulation

The inactivated vaccines were generated according to the method used in a previous study [16]. Briefly, 15 μg (1 dose) each of purified 146S antigens of SAT1 BOT 1/68-R, SAT2 ZIM 5/81-R, and SAT3 ZIM 4/81-R) were mixed with 10% aluminum hydroxide gel (Rehydragel® HPA; General Chemical, NJ, USA), to which 0.5 μg of saponin was added; 1 ml of the resultant solution in the form of water-in-oil-in-water with ISA206 (Seppic, France) was defined as a single dose [19].

### Nucleotide and amino acid similarities among the FMD SAT vaccine strains

The available nucleotide and amino acid sequences of the P1 region (VP4, VP2, VP3, and VP1) were compared with those of the SAT1 BOT 1/68, SAT2 ZIM 5/81, and SAT3 ZIM 4/81 vaccine strains, and their similarities were determined using the clone manager program (Professional Edition 9). The Genbank accession for similarity comparison of nucleotides and amino acid sequences was presented in the following order: SAT1 BOT 1/68 (AY593845, topotype III); Sat1-5sa iso13 (AY593842, topotype III); SAT1–1 bech (NC_011451, topotype III); SAT1 KEN/5/98 (DQ009721, topotype III); SAT1/NIG/4/15 (MF678826, topotype X); SAT2 ZIM 5/81 (EF134951, topotype II); SAT2 SAU/6/00 (AY297948, topotype VII); SAT2 7/83(AF540910, topotype VI); SAT2 RWA/02/01 (DQ009730, topotype VIII); SAT2 3Kenya-21 (AY593849, topotype X); SAT3 ZIM 4/81 (KX375417, topotype I); SAT3-2sa iso27 (AY593850, topotype I); SAT3 ZIM/05/91/3 (DQ009740, topotype III); SAT3 KNP 10/90/3 (AF286347, topotype I); SAT3 UGA/1/13 (KJ820999, topotype V).

### Immunization of pigs and cattle with the experimental vaccine

The ten-week-old pigs (*n* = 20) that were used for this study were inoculated with the experimental vaccine. Sera were collected until 10 weeks (0, 14, 28, 42, 56, and 70 day post-vaccination) after vaccination. Further, the five-month-old cattle (*n* = 5) that were used for this study were also inoculated with the experimental vaccine. Sera were collected until 20 weeks (0, 14, 28, 42, 56, 72, 84, 112, 140 day) after vaccination.

### Assessment of the challenge test after immunization of the pigs

The pigs were subjected to a virus challenge 28 days after immunization with the vaccines (15 μg/ml). The antibody titers determined by an immunological experiment were assessed using a virus neutralization test (VNT). These tests were conducted according to the previous methods [16] for virus challenge in the vaccination group. Briefly, the neutralizing antibody titers in the serum were measured using the VNT specified in the Manual of Diagnostic Tests and Vaccines for Terrestrial Animals of the World Organisation for Animal Health (OIE). Serum samples were collected from the animals after vaccination. The sera were heat-inactivated at 56 °C for 30 min. Following 1 h incubation in serial diluted sera and virus suspension, LF-BK cells were added to the plate and incubated for a period of 2–3 days. The neutralizing antibody titers were calculated as the log10 of the reciprocal antibody dilution to neutralize 100 50% tissue culture infective doses (TCID_50_) of the virus. The pigs vaccinated with inactivated SAT1, SAT2, or SAT3 were challenged with each homologous virus of three serotypes in the heel bulb, which is a region sensitive to FMDV, at 10^5^ TCID_50_/0.1 ml and observed for 2 weeks. After the challenge inoculation, the virus levels in nasal discharge and serum samples were monitored for 7 days by collecting the samples at one-day intervals. The FMDV viral RNA was identified by extracting the viral RNA from oral swab samples and quantitative real-time reverse transcription PCR (RT-PCR). The MagNapure 96 system (Roche, Germany) was used for the extraction of the viral RNA, and the quantitative real-time RT-PCR was conducted using the same method as in the previous experiment [16]. The clinical score was determined by the addition of points distributed as described below. The clinical observation was performed daily after the challenge. The clinical scores were calculated using the following criteria: (a) elevated body temperature of 40 °C (1 point), > 40.5 °C (2 points), or > 41 °C (3 points); (b) lameness (1 point); (c) hoof and foot vesicles (1–2 points per foot); and (d) snout, lips, and tongue vesicles (1 point for each affected area), for a maximum of 15 points.

## Results

### Characteristics of the FMD vaccine viruses

After the FMD SAT1, SAT2, and SAT3 viruses were recovered (Fig. 1a), each virus strain was examined using electron microscopy, and approximately 25-nm virus particles were identified (Fig. 1b). their surface-expressed antigens were examined by antigen ELISA, thereby confirming the expression of the structural proteins (Fig. 1c).

**Fig. 2:**
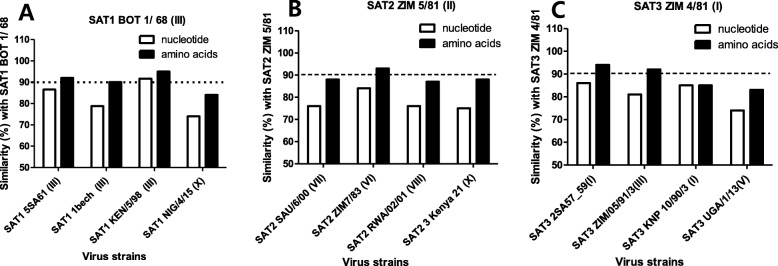
Genetic variations of SAT 1, SAT 2, and SAT 3 vaccine strains. **a**-**c** Similarity of the available P1 nucleotide and amino acids sequences (VP4, VP2, VP3, and VP1) with FMD vaccine strains in the SAT1, SAT2, and SAT3 virus; Genbank accession order: SAT1 BOT 1/68 (AY593845, topotype III); SAT1-5sa iso13 (AY593842, topotype III); SAT1–1 bech (NC_011451, topotype III); SAT1 KEN/5/98 (DQ009721, topotype III); SAT1/NIG/4/15 (MF678826, topotype X); SAT2 ZIM 5/81 (EF134951, topotype II); SAT2 SAU/6/00 (AY297948, topotype VII); SAT2 7/83(AF540910, topotype VI); SAT2 RWA/02/01 (DQ009730, topotype VIII); SAT2 3Kenya-21 (AY593849, topotype X); SAT3 ZIM 4/81 (KX375417, topotype I); SAT3-2sa iso27 (AY593850, topotype I); SAT3 ZIM/05/91/3 (DQ009740, topotype III); SAT3 KNP 10/90/3 (AF286347, topotype I); SAT3 UGA/1/13 (KJ820999, topotype V)

When the similarity of P1 was compared among the vaccine strains based on their surface antigens (Figs. 2a-c), the SAT1 vaccine strain—which belongs to topotype III—showed a 74%~ 91% nucleotide similarity and a 84%~ 95% amino acid similarity among topotypes III, and X, thereby indicating that its amino acid sequence showed higher similarity than its nucleotide sequence. The SAT2 vaccine strain, which belongs to topotype II, showed a 75%~ 84% nucleotide similarity and an 87%~ 93% amino acid similarity with topotypes II, VI, VII, and VIII. The SAT3 vaccine strain, which belongs to topotype I, showed a 74%~ 86% nucleotide similarity and an 83%~ 94% amino acid similarity with topotypes I, III, and V.

### Immunogenicity of the vaccines in cattle and pigs

**Fig. 3:**
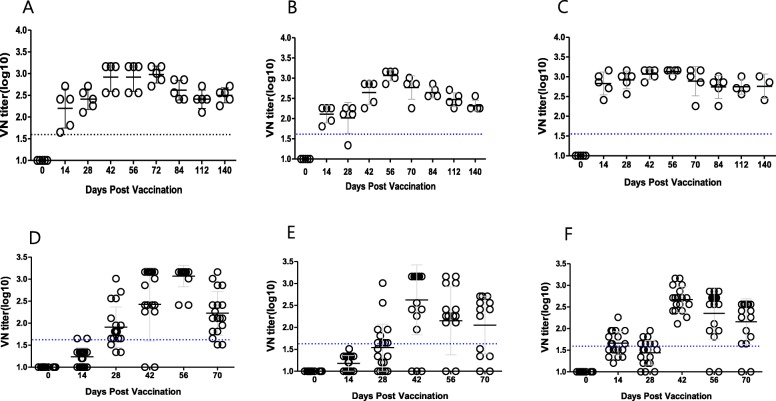
Immunogenicity in the pigs and cattle vaccinated with SAT vaccines. **a** Virus-neutralizing titers in the cattle (*n* = 5) vaccinated with SAT1 vaccine. **b** Virus-neutralizing titers in the cattle (*n* = 5) vaccinated with the SAT2 vaccine. **c** Virus-neutralizing titers in the cattle (*n* = 5) vaccinated with SAT3 vaccine. **d** Virus-neutralizing titers in the pigs (*n* = 20) vaccinated with SAT1 vaccine. **e** Virus-neutralizing titers in the pigs (*n* = 20) vaccinated with SAT2 vaccine. **f** Virus-neutralizing titers in the pigs (*n* = 20) vaccinated with SAT3. The dotted lines show 1.65 log10 virus-neutralizing (VN) titers (1:45). Vaccination in pigs and cattle was done two times at 0 and 28 days. The error bars are the standard deviation

In terms of immunogenicity of the SAT1, SAT2, and SAT3 strains in cattle, high and uniform levels of neutralizing antibodies were detected within 2 weeks, and these antibodies tended to persist for up to 140 days (Figs. 3a-c). In pigs, the antibody titers were elevated to a relatively high level, exceeding 1:45 (1.65 log_10_) on average only for the SAT1 strain in the fourth week after vaccination. In contrast, the antibody titers did not reach 1:45 on average for the SAT2 and SAT3 strains in the fourth week after vaccination. However, the antibody titers were elevated for all strains after the second vaccination, which persisted up to 70 days after vaccination (Figs. 3d-f). It must be noted that two pigs in SAT2 vaccinated group showed no reactivity.

### Protection in immunized pigs

**Fig. 4:**
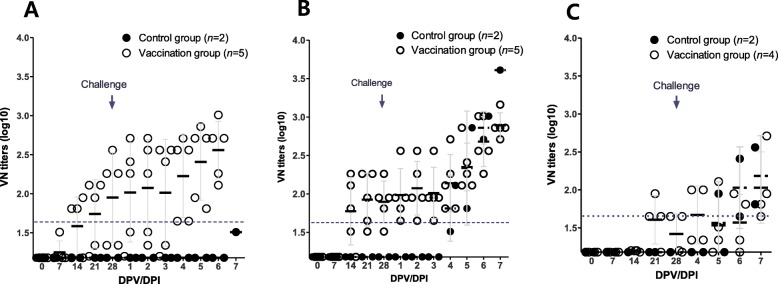
Immune responses against SAT viruses measured by a virus neutralization test in immunized pigs for the challenge test. Antibody responses by VN titers for SAT viruses in pigs at 0, 7, 14, and 28 days post-vaccination and 1~7 days post-challenge. **a** SAT1-BOT-R (*n* = 5). **b** SAT2-ZIM-R (*n* = 5). **c** SAT3-ZIM-R (*n* = 4). The dotted lines represent 1.65 log10 virus-neutralizing (VN) titers (1:45). The error bars are the standard deviation

**Fig. 5:**
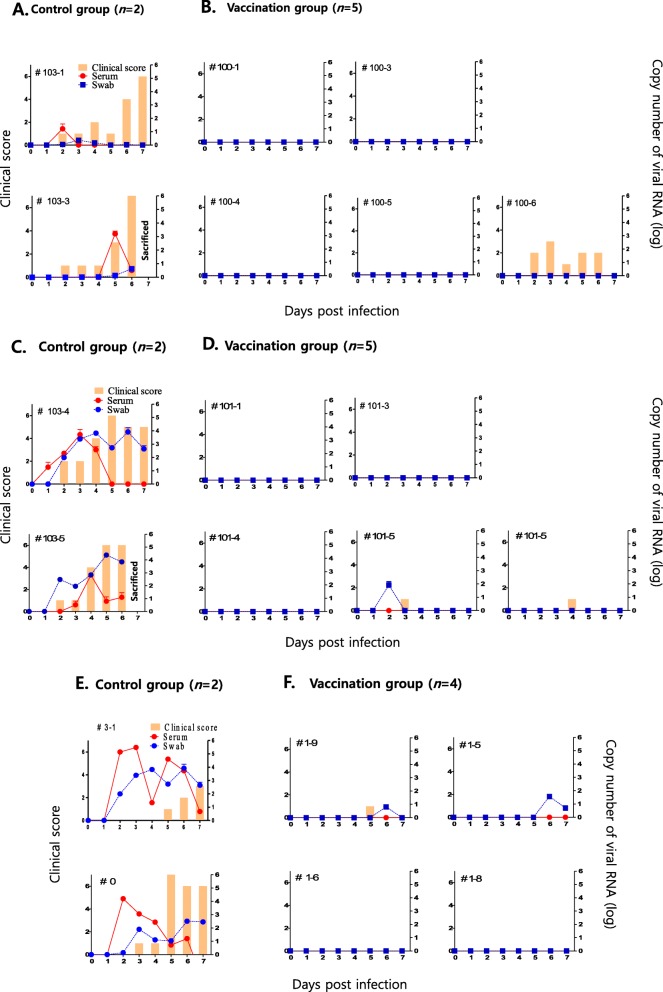
Clinical scores and virus shedding in the pigs immunized with the SAT1, SAT2 or SAT3 vaccine after the SAT1 BOT 1/68, SAT2 ZIM-5/81 or SAT3 ZIM 4/81 virus challenge. **a** Negative control group (*n* = 2) for SAT1 virus challenge. Control pig #103–3 was sacrificed at 7 day post-challenge (dpc). **b** SAT1 BOT-R-vaccinated group (*n* = 5), **c** Negative control group (*n* = 2) for SAT2 virus challenge, Control pig #103–5 was sacrificed at 7 dpc. **d** SAT2 ZIM-R-vaccinated group (*n* = 5), **e** Negative control group (*n* = 2) for SAT3 virus challenge. **f**. SAT3 ZIM-R-vaccinated group (*n* = 4)

In the animals immunized for the challenge test, high titers (approximately 1:100) of neutralizing antibodies were also inducted as part of the SAT1 challenge 28 days after the immunization (Fig. 4a). SAT2 also induced relatively uniform levels of neutralizing antibodies at titers as high as those induced by SAT1 (Fig. 4b); however, SAT3 induced relatively low levels of neutralizing antibodies (Fig. 4c). The challenge test confirmed that pig groups that were immunized with the vaccines were protected from SAT1, SAT2 or SAT3 wild-type virus challenge (Figs. 5a-f). In the immunized group, viremia and virus shedding were barely detected. However, pig #100–6 exhibited an elevated body temperature for a short period time 3~6 days after the challenge, although no symptoms specific to FMD were observed (Fig. 5b). In certain cases, mild lesions were observed at the injection site after the SAT2 or SAT3 challenge.

## Discussion

Outbreaks of SAT-type FMDV are historically restricted to Sub-Saharan Africa but have caused outbreaks in North Africa and the Middle East.

These viruses were separated into the Euro-Asiatic type and the South African type in the late seventeenth and early eighteenth centuries, with distinct origins [20]. Compared with the Euro-Asiatic type, the SAT viruses underwent relatively a large number of change in terms of structural proteins [21], but not so many changes were observed in non-structural proteins. Lee et al. [18] demonstrated that a new serotype vaccine strain could be developed by replacing the P1 gene of FMDV with that of the O1 Manisa virus, thereby confirming that gene switching between the seven serotypes does not affect the survival of the virus [18]. Based on these experimental results, it was possible to construct a new virus strain by including P1 from the vaccine strains, which has been widely used for vaccine production.

If the rate of change in the virus, as seen in the evolution of FMD viruses or the introduction of new viruses, outpaces vaccine development research, it will become more difficult to protect against the virus [22, 23]. In this respect, this study provided a framework for rapid development of new vaccines, proposing that the establishment of a more reliable method for vaccine development that surpasses the evolution rate of the virus can be a means to advance the vaccine research a step further [22, 23]. Thus far, the SAT serotype viruses are known to mainly infect cattle and African buffalos [24–26]. There are not many large pig farms in sub-Saharan Africa.

Pigs have not been seriously considered as the target of these viruses; thus, it is difficult to determine the effect of vaccines if the outbreak occurs in pigs. Therefore, the SAU/6/00 virus vaccine was developed against SAT2 and was tested in pigs to determine its protection capacity against a homologous virus. It was confirmed that this vaccine provided clinical protection up to 80%, which is similar to this study, with virus neutralizing (VN) titer of mean 2.5 log_10_ [1] by single vaccination. The SAT1 KNP/196/91 vaccine induced complete protection with VN titer of mean > 1.3 log_10_ against homologous challenge in pigs [11]. In the present study, whether wild-type, SAT1, SAT2, and SAT3 viruses can infect pigs was examined through a challenge test; it was found that pigs can be infected by these viruses. Further, the spreading of infectious viruses to Asia is considered possible [27, 28].

The outbreak of the SAT-type-mediated FMD is endemic to areas surrounding Africa and a rare event outside of Africa. The most representative international vaccine strains against SAT1-type FMD that is usually recommended by the FMD World Reference Laboratory of the OIE are the South Africa and Kenya strains—the Saudi Arabia (Eritrea) and Zimbabwe strains are recommended for SAT2 and the Zimbabwe strain is recommended for SAT3. The SAT serotypes can be divided into 5~14 topotypes in each serotype [29]. The minimum countermeasures for the influx of SAT viruses must be considered in preparation from the Middle East or other Asian regions. In terms of immunogenicity against SAT1, SAT2, and SAT3, high titers of neutralizing antibodies were detected in cattle within the short term, which persisted for the long term. In pigs, the antibody titers were elevated for all SAT types after the second vaccination, which persisted for up to 3 months. The same phenomenon was also observed in pigs that were immunized for the challenge test; it was found that despite the difference in antibody titers for different SAT types, all pigs were protected from the challenge, except for one pig which exhibited elevated body temperature following the SAT1 challenge without classical clinical signs. VN titers in cattle were revealed to be higher than that in pigs. Two pigs in SAT2 vaccination group showed no reactivity. The reason for non-reactivity in the pigs was not clear because of the experiment in the field.

This result confirms that the vaccines for the three SAT types provided enough immunity even in pigs and also provided protection against the virus challenge. In the event of an FMD outbreak caused by the introduction of an unvaccinated serotype, FMD can be controlled by disinfection, restricted animal movement, and destroying of infected animals; however, more effective measures can be implemented if efficacious vaccines are available. Although FMD outbreaks of SAT serotypes are believed to be very unlikely, it is still possible that the viruses are transmitted through intercontinental migration like African swine fever [30]; thus, careful preparations for such an event are necessary.

## Conclusions

Representative vaccine strains for SAT1, SAT2, and SAT3 serotypes of FMD were developed and their immunological reactivity for the protection of cattle and pigs was confirmed. With regards to the lack of evaluation of vaccine strains against SAT serotypes in pigs, the vaccine strains developed in this study are expected to be used as vaccines that can protect against FMD in the event of a future FMD outbreak in pigs in consideration of the situation in Asia.

## Data Availability

We did not share the raw data anymore because of having details in figures of this article.

## Abbreviations

ABSL-3: Animal biosafety level-3
APQA: Animal and Plant Quarantine Agency
BEI: Binary ethylenimine
BHK: Baby hamster kidney
BL-3: Biosafety level-3
ELISA: Enzyme-linked immunosorbent assay
FMD: Foot-and-mouth disease
FMDV: Foot-and-mouth disease virus
OD: Optical density
OIE: World Organisation for Animal Health
PBST: Phosphate buffered saline with tween
PCR: Polymerase chain reaction
RNA: Ribonucleic acid
RT-PCR: Reverse transcription PCR
SAT: South African Territories
TCID_50_: 50% tissue culture infective doses
TMB: Tetramethyl -benzidine
VN: Virus neutralizing
VNT: Virus neutralization test
